# Cachexia Phenotyping Through Morphofunctional Assessment and Mitocondrial Biomarkers (GDF-15 and PGC-1α) in Idiopathic Pulmonary Fibrosis

**DOI:** 10.3390/nu17172739

**Published:** 2025-08-24

**Authors:** Alicia Sanmartín-Sánchez, Rocío Fernández-Jiménez, Josefina Olivares-Alcolea, Eva Cabrera-César, Francisco Espíldora-Hernández, Isabel Vegas-Aguilar, María del Mar Amaya-Campos, Víctor José Simón-Frapolli, María Villaplana-García, Isabel Cornejo-Pareja, Ana Sánchez-García, Mora Murri, Patricia Guirado-Peláez, Álvaro Vidal-Suárez, Lourdes Garrido-Sánchez, Francisco J. Tinahones, Jose Luis Velasco-Garrido, Jose Manuel García-Almeida

**Affiliations:** 1 Department of Endocrinology and Nutrition, Son Espases University Hospital, 07120 Mallorca, Spain; jolivares@ssib.es (J.O.-A.); m.villaplana.garcia@gmail.com (M.V.-G.); 2 Department of Endocrinology and Nutrition, Virgen de la Victoria University Hospital, 29010 Malaga, Spain; isabel.mva13@gmail.com (I.V.-A.); mariadelmarac2@gmail.com (M.d.M.A.-C.); victorsimonfrapolli.med@gmail.com (V.J.S.-F.); isabelmaria_cornejo@hotmail.com (I.C.-P.); anaasanchez12@gmail.com (A.S.-G.); moramurri@gmail.com (M.M.); pguirado1991@gmail.com (P.G.-P.); alvarovidal1992@gmail.com (Á.V.-S.); lourdes.garrido@ibima.eu (L.G.-S.); fjtinahones@uma.es (F.J.T.); jgarciaalmeida@gmail.com (J.M.G.-A.); 3 Instituto de Investigación Biomédica de Málaga y Plataforma en Nanomedicina-IBIMA Plataforma BIONAND, 29010 Malaga, Spain; 4 Department of Medicine and Dermatology, Málaga University, 29016 Malaga, Spain; 5 Department of Endocrinology and Nutrition, Quironsalud Málaga Hospital, Av. Imperio Argentina, 29004 Malaga, Spain; 6 Department of Neumology, Virgen de la Victoria University Hospital, 29010 Malaga, Spain; evacabreracesar@gmail.com (E.C.-C.); jlvelascogarrido@hotmail.com (J.L.V.-G.); 7 Department of Neumology, Regional University Hospital, 29010 Malaga, Spain; fespildorahernandez@gmail.com; 8 Centro de Investigación Biomédica en Red Fisiopatología de la Obesidad y Nutrición (CIBEROBN), Carlos III Health Institute (ISCIII), University of Málaga, 29010 Malaga, Spain; 9 Instituto de Investigación Biomédica de Málaga y Plataforma en Nanomedicina-IBIMA Plataforma BIONAND, Heart Area, Victoria Virgen University Hospital, 29010 Malaga, Spain

**Keywords:** idiopathic pulmonary fibrosis, cachexia, sarcopenia, myosteatosis, GDF-15, PGC-1α, morphofunctional assessment, body composition, malnutrition, chronic pulmonary diseases

## Abstract

**Background/Objetives**: Idiopathic pulmonary fibrosis (IPF) is a progressive interstitial lung disease with poor prognosis. Nutritional disorders, particularly cachexia, significantly impact morbidity and mortality in IPF but remain under-investigated. This study aimed to characterize cachexia phenotypes in IPF through morphofunctional assessment (MFA) and to evaluate their prognostic relevance, including the role of mitochondrial biomarkers. **Methods**: In this prospective bicenter study, 85 IPF patients underwent MFA including bioelectrical impedance vector analysis (BIVA), nutritional ultrasound (NU), and T12-level computed tomography (T12-CT) for body composition. Functional and strength assessments included timed up and go test (TUG) and handgrip strength (HGS), respectively. Cachexia was defined by Evans’ criteria, Martin’s CT-based criteria, and our IPF-specific proposed definition. Serum GDF-15 and PGC-1α levels were also measured. **Results**: Cachexia prevalence varied by definition: 24.71% (Evans), 29.5% (Martin) and 42.4% (IPF Cachexia Syndrome). Cachectic patients showed significantly lower muscle mass, function, and quality (measured by reduced muscle attenuation at T12-CT), along with higher GDF-15 and lower PGC-1α levels. The presence of IPF Cachexia syndrome (HR 2.56; 95% CI, 1.08–6.07; *p* = 0.033), GDF-15 > 4412.0 pg/mL (HR 3.21; 95% CI, 1.04–9.90; *p* = 0.042) and impaired TUG (>8 s) (HR 3.77; 95% CI, 1.63–8.71; 0.002) were all independently associated with increased 24-month mortality. **Conclusions**: Cachexia is prevalent in IPF and showed strong concordance between the three diagnostic criteria. The IPF Cachexia syndrome, based on comprehensive morphofunctional phenotyping, demonstrated superior discriminatory capacity. The addition of mitochondrial biomarkers may improve early detection and support personalized interventions to improve patient outcomes.

## 1. Introduction

Idiopathic pulmonary fibrosis (IPF), known as a chronic and progressive lung disease due to irreversible interstitial fibrosis of the pulmonary parenchyma [1], is associated with poor prognosis [1,2,3], even though new antifibrotic medications have been introduced [3,4,5]. Its devastating prognosis is partly attributed to patients’ comorbidities [2,6,7,8,9,10,11], such as diabetes, cardiovascular diseases, systemic and pulmonary hypertension, or gastroesophageal reflux. Among these, nutritional disorders are becoming increasingly relevant, as they have been linked to all-cause hospitalization [12], increased ICU length of stay, lower median survival [13,14] and mortality [2,3,12,13,15,16], as well as to a higher incidence of disability, falls, and poorer quality of life in IPF [14,16,17,18].

Patients with chronic respiratory diseases, particularly chronic obstructive pulmonary disease (COPD), are susceptible to malnutrition through several pathophysiological mechanisms, including elevated energy expenditure due to increased work of breathing, systemic inflammatory cascade activation, and tissue hypoxia-induced metabolic dysfunction [18,19]. IPF and COPD share common mechanisms underlying nutritional deterioration, although IPF’s accelerated progression may lead to a more rapid nutritional compromise [20].

Of all nutritional complications, cachexia represents the most severe phenotype, defined as the final consequence of various unresolved diseases, including infections, chronic inflammatory conditions, and cancers [21], and described as “a multifactorial syndrome characterized by an ongoing loss of skeletal muscle mass (with or without loss of fat mass) that cannot be fully reversed by conventional nutritional support and leads to progressive functional impairment” [22]. With an estimated annual death rate of 2 million people worldwide, cachexia is one of the main contributors to human morbidity and mortality [23], although it is often underdiagnosed.

Although the term “pulmonary cachexia” refers to cachexia linked to chronic lung diseases, it is most commonly reported in patients with COPD [24]. Evidence on cachexia in COPD is growing [25,26,27,28,29,30], and it is recognized as an independent and significant mortality risk factor [26,29]. Cachexia prevalence in COPD have been reported from 4.6% in a cohort of 2739 stable outpatients [30] to 7.9% in a cohort of 1,446,431 hospitalized COPD patients [29], who experienced significantly more inpatient complications, longer mean lengths of stay and higher in-hospital mortality [29]. However, studies addressing cachexia in IPF remain extremely limited.

The different causes of cachexia suggest the existence of subtypes [21,25], driven by inflammatory mechanisms involving tumor necrosis factor-alpha (TNF-α) [31], interleukin-6 and interleukin-1 [32], chemokine CCL2 [33], lipocalin-2 [34], or macrophage activation [35], along with imbalances in molecules that maintain tissue homeostasis. Mitochondrial oxidative stress has also been associated with greater muscle atrophy and lower levels of peroxisome proliferator activated receptor gamma coactivator 1-alpha (PGC-1α) [36], as well as altered expression of transcription factors involved in lipid metabolism [37], insulin resistance [38] and appetite and nutrient intake suppression through growth differentiation factor 15 (GDF-15) [39,40]. In addition, vitamin D deficiency has been associated with impaired muscle function, sarcopenia, and enhanced muscle inflammation in chronic inflammatory conditions, which may further contribute to multifactorial pathophysiology of cachexia [41].

Cachexia is preventable and potentially reversible. Nonetheless, its reversal remains a major challenge. Several diagnostic criteria have been proposed: the Fearon definition [22], widely used in cancer cachexia; the Evans criteria [42], applicable to chronic diseases and regarded as the most widely accepted criteria for clinical cachexia; and more recent approaches incorporating morphofunctional assessment through computed tomography (CT), such as the Martin criteria [43].

Therefore, the main aim of this study is to compare three definitions of cachexia—Evans, Martin and the new IPF Cachexia Syndrome—focusing on the differences in patients’ characteristics according to each definition. Additionally, we aim to assess the prognostic value of these definitions using morphofunctional assessment in which body composition from Bioelectrical Impedance Vector Analysis, nutritional ultrasound and CT thoracic scans (CT data was available for 61 patients of the cohort) are included, alongside mitochondrial biomarkers.

## 2. Materials and Methods

### 2.1. Study Design and Patient Selection

We conducted an observational, prospective, bicenter study at Virgen de la Victoria University Hospital (Málaga) and the Regional University Hospital of Málaga, where patients were assessed as part of routine clinical follow-up in nutrition and pneumology units between 2022 and 2023. All patients included had to be adults (age ≥ 18 years) already diagnosed of IPF according to ATS/ERS/JRS/ALAT guidelines [2] and fullfill the informed consent for participation in the research. Patients were excluded if they presented contraindications to body composition analysis or had a life expectancy < 3 months (Figure S1).

The study was approved by the regional ethics committee (approval number: 1743-N-21) on 5 April 2022 and made in accordance with the Declaration of Helsinki.

### 2.2. Morphofunctional Assessment

Nutritional status was assessed using an integrative morphofunctional approach (body composition along with functional measurements) [44,45]. For body composition we used three techniques. First, Bioelectrical Impedance Vector Analysis (BIVA) [46,47], using a standardized protocol with an AKERN NutrilabTM tetrapolar device. Standardized phase angle values (SPhA) were derived using age- and sex-adjusted reference equations.

Secondly, Nutritional Ultrasound (NU) [48], performed by trained clinicians to evaluate rectus femoris muscle (cross-sectional area, circumference, thickness and subcutaneous adipose tissue) and abdominal fat compartments.

Finally, Thoracic CT at the T12 vertebral level (T12CT), the best vertebra level in thoracic CT for body composition [13,49,50,51,52,53,54,55] which, only if available a CT scan performed within ±3 months of the nutritional assessment (61 patients of our cohort), was analyzed using FocusedON^®^ software (pilot version, ARTIS Development, Barcelona, Spain) [49,56,57,58], https://focusedon.es (accessed on 20 June 2025), an automatic tissue segmentation tool developed by ARTIS Development and based on artificial intelligence. We quantify skeletal muscle area (SMA), skeletal muscle index (SMI), intramuscular adipose tissue (IMAT), subcutaneous (SAT), and visceral adipose tissue (VAT).

For functional measurements, muscle strength was assessed using the handgrip strength (HGS) test, following European Working Group on Sarcopenia in Older People 2 (EWGSOP2) recommendations [59]. Physical performance was evaluated using the Timed Up and Go (TUG) test [60]. All patients underwent pulmonary function testing, including forced vital capacity (FVC) and diffusion capacity of the lung for carbon monoxide (DLCO), according to standard guidelines.

### 2.3. Diagnostic Criteria for Cachexia

Cachexia was diagnosed based on Evans criteria, which require unintentional weight loss ≥ 5% over 12 months (or BMI < 20 kg/m^2^), plus at least three of the following: reduced food intake, low fat-free mass index (<17 kg/m^2^ in men or <15 kg/m^2^ in women), fatigue, low muscle strength (<27 kg for men or <16 kg for women), and elevated inflammatory markers (e.g., C-reactive protein (CRP) > 5 mg/L) [42].

It was also defined according to the Martin et al. criteria, which combine unintentional weight loss, sarcopenia and myosteatosis. Sarcopenia (defined by low SMI) and myoesteatosis (defined by elevated IMAT) were originally measured by CT at the L3 level. In our study, muscle was assessed at the T12 level, as this was the available CT slice. In the original Martin study, cut-offs values for reduced muscle mass and muscle density were based on survival data, identifying the thresholds most associated with poor prognosis. Following the same concept, we applied the criteria at T12 level using the thresholds already published in IPF patients at T12CT where a SMI at T12CT level (SMI_T12CT) ≤ 28.8 cm^2^/m^2^ [49] and an IMAT > 15.25% [56] were associated with poorer survival. All three components were required to classify a patient as cachectic [43].

We also applied a set of diagnostic criteria adapted from Evans et al. and Martin et al. incorporating morphofunctional parameters validated in our cohort (IPF Cachexia Syndrome). IPF Cachexia Syndrome was diagnosed when the following three conditions were present: unintentional weight loss greater than 5% of habitual body weight over the previous six months; evidence of systemic inflammation, defined by a CRP concentration > 5 mg/L; and changes in muscle mass (lower quantity or quality). Muscle mass was assessed using T12CT, with low muscle mass defined as a skeletal muscle index (SMI) < 24.8 cm^2^/m^2^, based on cut-offs values previously established in our population [49], or alternatively by BIVA, using EWGSOP2 cut-off points for appendicular skeletal muscle mass index: <7 kg/m^2^ for men or <5.5 kg/m^2^ for women, as these are the most widely standardized thresholds in the literature for diagnosing low muscle mass [59]. Muscle quality was determined through the quantification of myosteatosis, calculated using the formula: IMAT% = IMAT (cm^2^)/[SMA (cm^2^) + IMAT (cm^2^)] × 100 [53]. Myosteatosis was considered present when IMAT% exceeded 15%, in accordance with thresholds validated in our prior work [56]. In addition, to facilitate clinical applicability, we generated an optimal cut-off point of 36.46 HU for muscle attenuation, which better discriminated individuals with myosteatosis (Figure S2). Patients who met all three criteria were classified as cachectic.

### 2.4. Mitocondrial Biomarkers

As part of the biochemical assessment, serum levels of GDF-15 and PGC-1α were measured. GDF-15 was included as a marker of inflammation-related anorexia and nutrient intake suppression [39], while PGC-1α was selected due to its role in mitochondrial function and muscle metabolism [36]. Both biomarkers were analyzed and explored as potential indicators of cachexia phenotype.

### 2.5. Statistical Analysis

Descriptive statistics were used to summarize clinical and morphofunctional variables. Continuous data was reported as mean ± standard deviation (SD), and categorical variables as frequencies (%). Comparisons between cachectic and non-cachectic patients were performed using Student’s *t*-test or Mann–Whitney U test for continuous variables and χ^2^ or Fisher’s exact test for categorical variables.

ROC curves determined optimal myosteatosis and biomarker cut-offs by Youden index. Survival was evaluated using Kaplan–Meier curves and log-rank test. To identify independent predictors of survival, multivariable Cox regression was performed, adjusting for age, sex, and BMI, providing hazard ratios (HR) with 95% confidence intervals (CI). Additionally, multivariable logistic regression was applied to evaluate mortality predictors, reporting odds ratios (OR) and 95% CI. A significance level was set at *p* < 0.05 for all statistical tests. Significance was set at *p* < 0.05 and all analyses were performed in JAMOVI software (version 2.3.28; The jamovi project, Sydney, Australia).

## 3. Results

### 3.1. Morphofunctional and Mitocondrial Biomarkers Characteristics Between Cachectic and Non-Cachectic According to Different Cachexia Criteria

A total of 85 patients with idiopathic pulmonary fibrosis were included, of whom 21 (24.7%) met the Evans criteria for cachexia. Compared to the non-cachectic group (*n* = 64), these patients showed significantly lower values in weight (74.03 ± 9.56 vs. 80.71 ± 12.99 kg; *p* = 0.033), BMI (26.82 ± 3.68 vs. 27.90 ± 3.38 kg/m^2^; *p* = 0.217), fat-free mass (FFM) (51.00 ± 6.43 vs. 56.30 ± 7.43 kg; *p* = 0.004), body cell mass (BCM) (23.70 ± 4.88 vs. 26.59 ± 5.03 kg; *p* = 0.024), and total body water (TBW) (38.12 ± 4.99 vs. 42.04 ± 6.05 L; *p* = 0.009). In addition, although not statistically significant, relevant reductions were observed in SMI_T12CT (24.09 ± 6.66 vs. 27.00 ± 6.90 cm^2^/m^2^; *p* = 0.142), RF-CSA (3.02 ± 0.69 vs. 3.45 ± 1.06 cm^2^; *p* = 0.155), and RF *Y*-axis (1.02 ± 0.22 vs. 1.15 ± 0.29 cm; *p* = 0.076). Regarding molecular markers, non cachectic patients presented slightly higher GDF-15 concentrations (4156.03 ± 2800.27 vs. 3943.80 ± 2035.28 pg/mL; *p* = 0.782) and PGC-1α expression (4.43 ± 2.59 vs. 3.79 ± 1.78; *p* = 0.360) (Table 1).

A total of 61 patients were analyzed using the proposed cachexia criteria adapted from Martin, with 18 patients (29.5%) classified as cachectic (Table 2). Compared to the non-cachectic group (*n* = 43), cachectic patients showed significantly lower values in BIVA parameters, such as phase angle (Pha) (4.34 ± 0.58 vs. 5.11 ± 0.72; *p* = 0.001), FFM (51.09 ± 6.72 vs. 57.12 ± 6.86 kg; *p* = 0.003), BCM (22.38 ± 3.57 vs. 27.89 ± 4.89 kg; *p* = 0.001), and TBW (38.27 ± 5.21 vs. 42.45 ± 5.38 L; *p* = 0.007) and nutritional index (673.82 ± 99.95 vs. 809.16 ± 180.84; *p* = 0.001). RF-CSA (2.75 ± 0.63 vs. 3.57 ± 1.04 cm^2^; *p* = 0.003), and RF-Y axis (1.01 ± 0.18 vs. 1.19 ± 0.32 cm; *p* = 0.018)from NU and SMI_T12CT (20.86 ± 4.28 vs. 28.42 ± 6.59; *p* = 0.001) and Muscle_HU_T12CT (35.22 ± 6.28 vs. 40.62 ± 7.06; *p* = 0.007) from T12CT were also significantly lower in the cachectic group. HGS was lower in the cachectic group (29.22 ± 9.13 vs. 34.77 ± 10.34 kg), with a borderline significance (*p* = 0.053). Serum levels of GDF-15 were significantly elevated in the cachectic group (5013.55 ± 3021.27 vs. 3552.46 ± 1380.68; *p* = 0.027), while PGC-1α tended to be lower (3.35 ± 2.37 vs. 4.79 ± 2.50; *p* = 0.097).

A total of 85 patients with IPF were evaluated, of whom 36 (42.4%) met the diagnostic criteria for cachexia based on the proposed IPF Cachexia Syndrome. Compared to the non-cachectic group (*n* = 49), these patients showed no significant differences in body weight (78.38 ± 13.68 vs. 79.56 ± 11.71 kg; *p* = 0.670), height (167.58 ± 9.06 vs. 169.96 ± 7.12 cm; *p* = 0.179), or BMI (27.82 ± 3.75 vs. 27.50 ± 3.28 kg/m^2^; *p* = 0.674). Fat-free mass (53.29 ± 7.33 vs. 56.23 ± 7.48 kg; *p* = 0.075), body cell mass (24.52 ± 4.41 vs. 26.87 ± 5.41 kg; *p* = 0.036), and total body water (39.70 ± 5.77 vs. 42.08 ± 6.06 L; *p* = 0.072) were all lower in the cachectic group, with BCM reaching statistical significance. Despite higher fat mass in cachectic individuals (25.09 ± 9.30 vs. 23.33 ± 7.06 kg; *p* = 0.325), the phase angle was slightly reduced (4.66 ± 0.65 vs. 4.95 ± 0.82; *p* = 0.094), and the nutritional index showed significantly lower values (707.92 ± 164.94 vs. 799.88 ± 150.07; *p* = 0.009). Regarding NU parameters, cachectic patients showed a significantly reduced RF-CSA: 3.03 ± 0.88 vs. 3.58 ± 1.02 cm^2^; *p* = 0.013) and lower RF-Y axis thickness (1.05 ± 0.23 vs. 1.17 ± 0.30 cm; *p* = 0.056). From a biochemical perspective, GDF-15 levels were slightly higher in the cachectic group (4187.15 ± 2344.46 vs. 4046.19 ± 2805.25 pg/mL; *p* = 0.472), while PGC-1α expression was lower (3.79 ± 2.12 vs. 4.59 ± 2.57; *p* = 0.163), although neither reached statistical significance (Table 3).

The prevalence and overlap of nutritional and body composition alterations among patients with idiopathic pulmonary fibrosis were examined by deconstructing the individual components of three established and proposed cachexia definitions. As shown in Table 4, the analysis captures the frequency of weight loss, inflammation, and low muscle mass or quality across the different diagnostic frameworks (Evans, Martin, and IPF-specific criteria), providing a comprehensive overview of the heterogeneity in nutritional phenotypes within this population.

### 3.2. Correlation Between Biomarker, Morphofunctional Parameters and Cachexia Criteria

**Figure 1:**
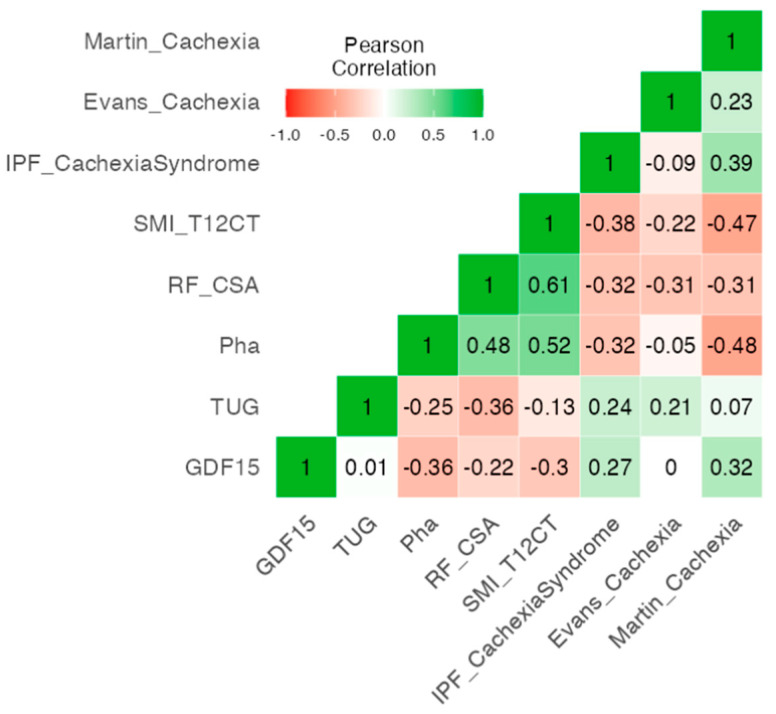
Correlation heatmap of morphofunctional and biochemical variables included in the IPF cachexia criteria. Abbreviations: IPF, idiopathic pulmonary fibrosis; SMI_T12CT, computed tomography at T12 level; RF_CSA, Cross-Sectional Area of the Rectus Femoris; Pha, Phase Angle; TUG, Timed Up an Go Test; GDF15, Growth Differentiation Factor 15.

Correlation analysis revealed inverse associations between GDF15 and morphofunctional parameters, including Pha (r = −0.36), and RF-CSA (r = −0.22) and SMI_T12CT (r = −0.3), suggesting that higher GDF15 levels are linked to diminished cellular integrity and muscle quality. Conversely, GDF15 showed positive correlations with the presence of cachexia when defined by IPF Cachexia Syndrome (r = 0.27) and Martin (r = 0.32). These cachexia classifications, in turn, exhibited negative correlations with Pha, RF-CSA and SMI_T12CT. In contrast, TUG correlates negatively with morphofunctional parameters and positively with the different criteria, demonstrating that function is somewhat important in this group of patients. The internal consistency of the IPF cachexia construct was acceptable, with a McDonald’s omega (ω) of 0.767, supporting the coherence and reliability of the selected variables in representing the syndrome (Figure 1).

### 3.3. Cut-Off Points for Parameters of Cachexia Syndrome

To define a population-specific cut-off point for muscle attenuation, we applied a ROC curve analysis based on Salhöfer’s criteria for myosteatosis [53].To facilitate clinical applicability, we using muscle attenuation values at the T12 vertebral level (Muscle_HU_T12CT), the optimal cut-off point identified was 36.46 HU. This cut-off provided an area under the curve (AUC) of 0.853 (95% CI 0.751–0.955, *p* < 0.001), with a sensitivity of 70.6%, a specificity of 92.6%, a positive predictive value of 92.3% and a negative predictive value of 71.4% (Figure S2). This result supports the use of this cut-off point as a reliable indicator of myosteatosis in our cohort of IPF patients, with excellent discriminative ability.

**Figure 2:**
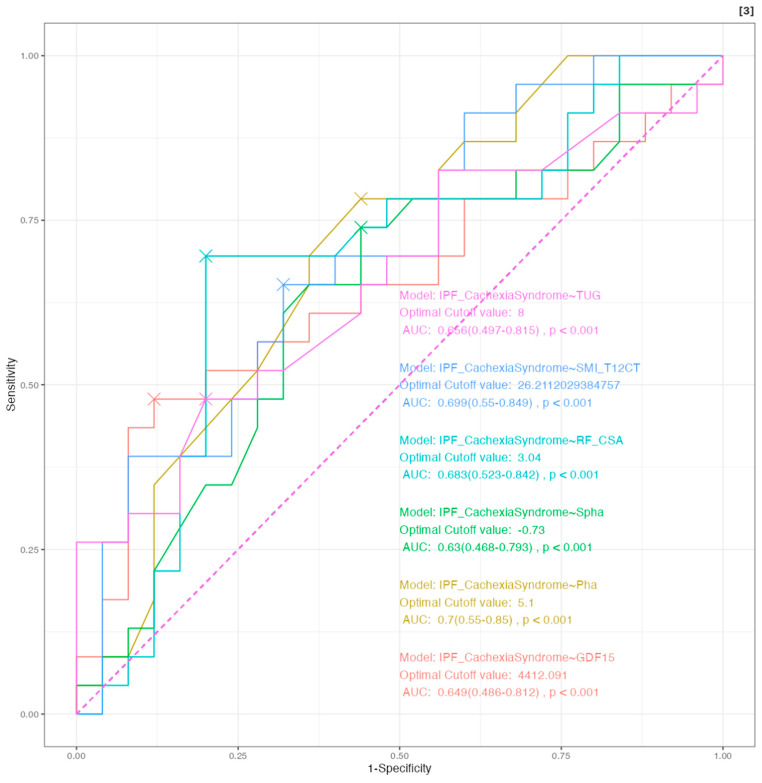
Multiple ROC curve analysis of morphofunctional and biochemical variables for the detection of cachexia according to the IPF-specific criteria. Abbreviations: IPF, idiopathic pulmonary fibrosis; SMI_T12CT, computed tomography at T12 level; RF_CSA, Cross-Sectional Area of the Rectus Femoris; Pha, Phase Angle; Spha, Standardized Phase Angl; TUG, Timed Up an Go Test; GDF15, Growth Differentiation Factor 15; AUC, area under the curve.

Using the cachexia definition generated specifically for this IPF cohort (IPF Cachexia Syndrome), we performed a ROC curve analysis to identify the most sensitive and specific cut-off points for various morphofunctional and biochemical parameters, which can be seen in Figure 2 and Figure S3. This approach aimed to provide clinically applicable thresholds that allow for early detection and intervention in cachexia. The cut-off values displayed in Table 5 represent practical diagnostic reference points derived from this population, enhancing the precision of cachexia identification based on structural, functional, and metabolic markers.

### 3.4. Survival Analysis

**Figure 3:**
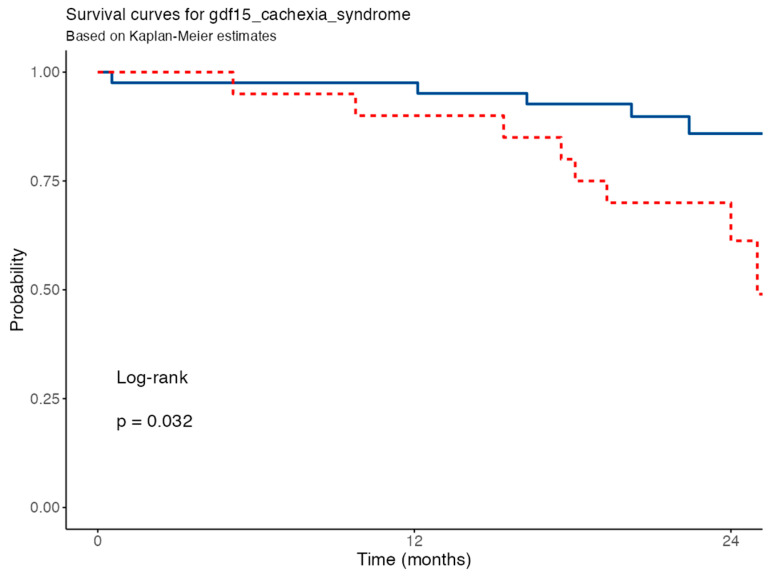
Survival curve for GDF15 levels. GDF15, growth differentiation factor 15. Blue solid line for patients with lower GDF15 concentrations, red dashed line for patients with higher levels.

Kaplan–Meier survival analysis revealed a significant association between elevated GDF15 levels (according to the IPF Cachexia Syndrome) and increased 24-month mortality (log-rank *p* = 0.032). Patients with high GDF15 concentrations had a significantly shorter survival compared to those with lower levels. In the univariable Cox regression model, elevated GDF15 was associated with a hazard ratio (HR) of 3.21 (95% CI: 1.04–9.90; *p* = 0.042), indicating over a threefold increased risk of mortality in this subgroup (Figure 3).

**Figure 4:**
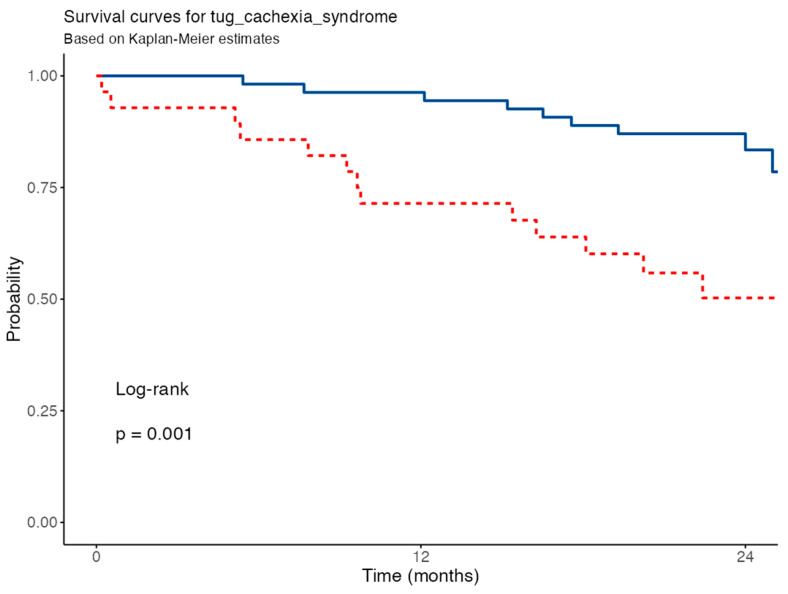
Survival curves for Time up and go test. Tug, Timed Up and Go test. Blue line for patients with normal TUG, red line for patients with impaired TUG.

Survival analysis based on TUG stratified by the IPF Cachexia Syndrome showed a significantly reduced survival in patients with impaired functional performance (TUG above the defined threshold), with a log-rank *p*-value of 0.0015. In the univariable Cox regression model, patients in the high TUG group had a hazard ratio (HR) of 3.77 (95% CI: 1.63–8.71; *p* = 0.002), indicating a more than threefold increased risk of mortality over 24 months compared to those with better functional mobility (Figure 4).

**Figure 5:**
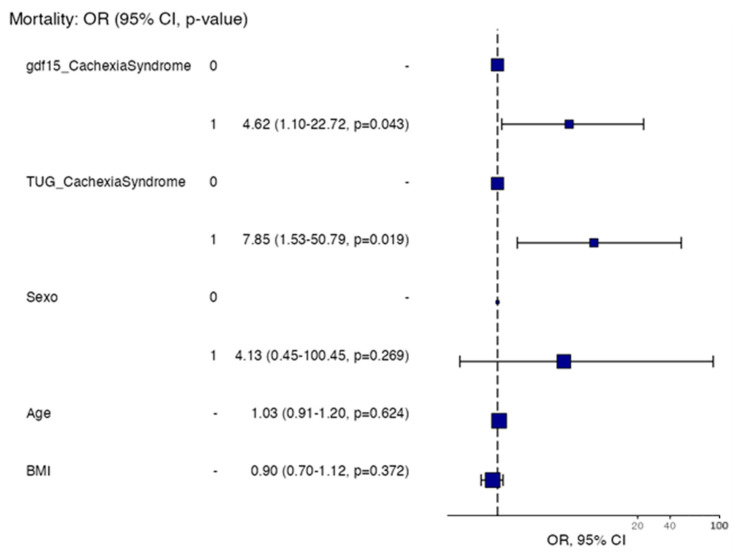
Odds ratio (OR) analysis for the relationship between selected variables and the presence of IPF Cachexia Syndrome. Variables include GDF15, TUG, Sex, Age, and BMI. Abbreviatures: GDF15: Growth Differentiation Factor 15; TUG: Timed Up and Go; BMI: Body Mass Index; CI: Confidence Interval.

Multivariable logistic regression analysis identified elevated GDF15 and impaired functional performance (TUG) as independent predictors of mortality in patients with idiopathic pulmonary fibrosis. Patients with high GDF15 levels had significantly increased odds of mortality (OR multivariable = 4.62 (1.10–22.72, *p* = 0.043)), and those with a pathological TUG result also showed a higher risk (OR multivariable = 7.85 (1.53–50.79, *p* = 0.019) (Table 6). Neither sex, age, nor BMI showed significant associations in the multivariable model (Figure 5).

**Figure 6:**
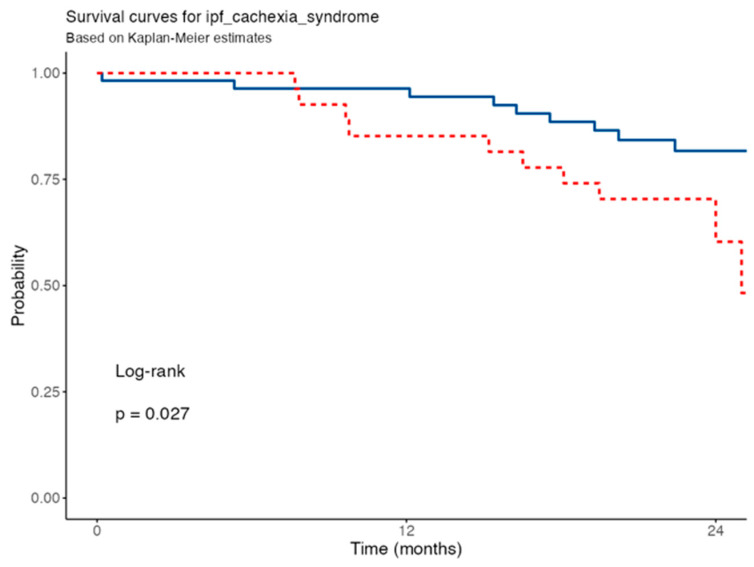
Survival curve for IPF Cachexia Syndrome. IPF: Idiopathic Pulmonary Fibrosis. Blue solid line for non-cachectic patients, red dashed line for cachectic patients according to IPF cachexia syndrome criteria.

The presence of IPF cachexia syndrome was significantly associated with lower survival. In the univariate Cox regression model, patients classified as cachectic according to our proposed criteria had a 2.56-fold higher mortality risk compared to non-cachectic individuals (HR = 2.56, 95% CI 1.08–6.07, *p* = 0.033). Kaplan-Meier survival analysis further illustrated this impact (Figure 6): the probability of survival at 12 months was 96.4% in non-cachectic patients versus 85.2% in cachectic individuals. Notably, the difference extended to 24 months, with survival decreasing to 60.3% (95% CI 40.9–89.0%) in the cachexia group versus 81.7% (95% CI 71.4–93.4%) in the non-cachectic group. These results were statistically significant, as confirmed by the log-rank test (*p* = 0.027), indicating that the presence of cachexia significantly shortens the survival time of IPF patients.

**Figure 7:**
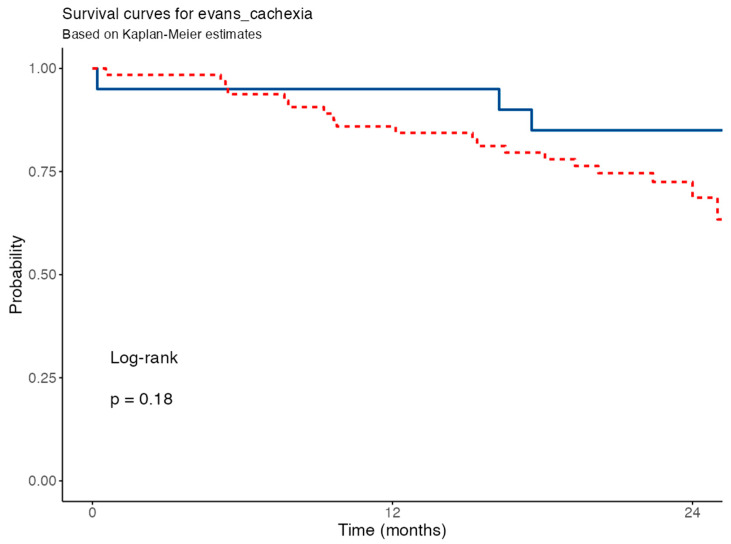
Survival curve for Evans’ criteria. Blue solid line for non-cachectic patients, red dashed line for cachectic patients according to Evans’ criteria.

Kaplan–Meier survival analysis comparing patients classified as cachectic vs. non-cachectic according to Evans’ criteria can be observed in Figure 7. No significant difference in survival was observed between groups (log-rank *p* = 0.191). In the univariable Cox model, Evans-defined cachexia was not associated with increased mortality (HR = 0.44; 95% CI: 0.13–1.50; *p* = 0.191).

**Figure 8:**
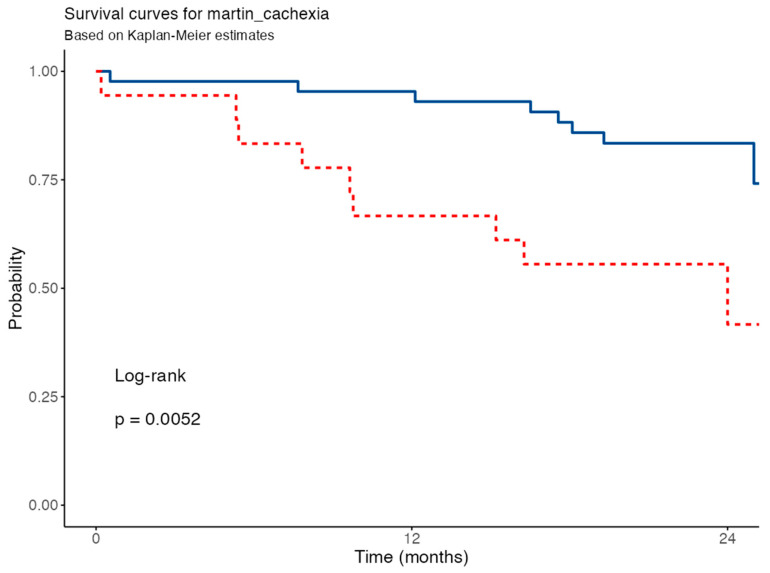
Survival curve for Martin’s criteria. Blue solid line for non-cachectic patients, red dashed line for cachectic patients according to Martin’s criteria.

Patients meeting Martin’s criteria showed significantly reduced survival (log-rank *p* = 0.009) in the Kaplan–Meier survival analysis comparing patients classified as cachectic vs. non-cachectic according to Martin’s CT-based criteria (Figure 8). In the univariable Cox model, Martin-defined cachexia was associated with a higher mortality risk (HR = 3.58; 95% CI: 1.38–9.33; *p* = 0.009).

Evans’ clinical definition did not stratify prognosis in our cohort, in contrast, CT-based definitions (Martin and the IPF Cachexia Syndrome) identified cachectic patients with significantly poorer survival.

## 4. Discussion

To the best of our knowledge, this is the first study focused on cachexia in IPF. While some studies mention it, such as Suzuki et al., they focus only on muscle mass loss without using cachexia diagnostic criteria, or those that focus exclusively on demonstrating associations between weight loss severity and mortality [2,61], with some patients not responding to nutritional management, without even mentioning cachexia as such.

There are a few articles from similar IPF patient samples to ours, discussing the importance of body composition for nutritional phenotyping [56,62], since changes in body composition are not only part of the diagnosis of malnutrition/sarcopenia/cachexia, but also an aggravating factor [45,62,63,64,65]. While Faverio et al. used criteria like Evans, no patients met these criteria. To date, no article has incorporated three types of cachexia criteria (in which were included the most validated, Evans’ criteria), nor any article focused on cachexia and IPF that includes three body composition techniques (T12-CT, NU, BIVA) with good correlation between them, and finally, no article that additionally includes mitochondrial markers showing good correlation with the MFA cachexia parameters.

Cachexia, a systemic wasting condition, is considered a late manifestation of multiple chronic diseases [21,42] and represents a complex metabolic syndrome associated with underlying illness infrequently identified and rarely treated [23,27]. Given that the underlying mechanisms causing cachexia are not well understood and its inherent poor prognosis represents metabolic failure in patients, cachexia remains a relatively uncommon condition for which there is no universally agreed-upon definition, making its diagnosis challenging [23]. Furthermore, its prevalence ranges from 5% to 15% in COPD or heart failure [66] to 60–80% in advanced cancer [66,67,68]. However, there are no epidemiological studies in IPF patients to estimate prevalence. In our cohort, we estimated a prevalence between 24.7–42.4% depending on the criteria used.

Considering that IPF mortality is estimated between 37–60% at 5 years from diagnosis [1,3,13,69], and without antifibrotic treatment reaches 50–70% at 3 years [69,70], and that loss of weight already occurs in 30% of IPF patients ≥ 5% in the first year [61], while in COPD it is 10–30% over 5 years [71,72,73,74] with acceptable reversibility when detected early [26,28], and given that IPF behaves similarly to many cancers [75,76,77] with equal or greater mortality than some malignancies [3,75], this could explain why the accelerated metabolic wasting leads to higher cachexia prevalence that may already be irreversible at detection.

Regarding cachexia diagnostic criteria types, although Fearon criteria [22] are well-known, we did not apply them to our sample as they are cancer-specific, while Evans criteria can be applied to all advanced chronic diseases. In our cohort Evans criteria showed up in 24.71% of total IPF patients, while Martin criteria only could be applied to those with available CT scans, even though they demonstrated a higher rate to Evans’ (29.5%). Martin criteria deserve special mention as they represent the first diagnostic criteria to incorporate myosteatosis, demonstrating that fulfilling these criteria is associated with significantly higher mortality by a factor of 4.3 (median survival 8.4 months vs. 28.4 months in non-cachectic patients) [43]. However, these criteria were developed primarily in lung cancer patients (78% of the cohort) [43], and we believe they may not be as sensitive as those we propose for IPF patients. Therefore, we advocate that in certain diseases like IPF, which present unique characteristics and a much more rapid and severe course, more sensitive criteria such as those we propose (IPF cachexia criteria, 42.4% of our sample) could potentially not be missing patients with early metabolic compromise who could benefit from timely intervention. Evans’ clinical definition did not stratify prognosis in our cohort, whereas both CT-based definitions (Martin’s criteria and the IPF Cachexia Syndrome) identified cachectic patients with significantly poorer survival. This underscores the prognostic value of integrating CT-derived muscle quality and quantity into cachexia definitions, particularly in IPF, where disease progression is rapid and metabolic deterioration is marked.

Our results demonstrate that the IPF Cachexia Syndrome is a strong prognostic indicator of survival in patients with IPF. Specifically, 24-month survival in patients classified as cachectic was markedly lower compared to non-cachectic patients (60.3% vs. 81.7%). This divergence was already evident at 12 months, with survival probabilities of 85.2% (cachexia group) versus 96.4% (non-cachexia group). These data highlight the progressive impact of metabolic deterioration in this population and support the utility of our proposed criteria for early identification of high-risk patients. While previous studies in COPD cohorts have shown a similar link between cachexia and mortality [24,25,26,28,29,30,68,71,72,73,78], few have evaluated this relationship in IPF [56,62,63], and none have done so using a multidimensional construct integrating weight loss, inflammation, and altered muscle quantity and quality. The findings underscore the importance of morphofunctional phenotyping in IPF and provide a rationale for incorporating cachexia assessment into routine prognostic evaluation.

Body composition integrating T12CT in these patients represents an opportunity, as CT scans are routinately performed during clinical follow-up, and T12 is the optimal vertebra for thoracic CT scans, being the nearest vertebra to L3 (known as the best single-slice CT for body composition [54,79] and showing high correlation with L3 [50,51,52,55], unlike L1 which is not visualized in most thoracic CT scans [49,80,81].

It is demonstrated that low muscle mass, as assessed by T12-CT, is associated with greater disease severity and increased mortality risk in IPF patients [49], also described in COPD patients [65,80]. Nonetheless, the opportunity we can exploit through CT is not only to evaluate quantity but quality, which is increasingly demonstrated to be equally or even more important.

Myoesteatosis, characterized by fat accumulation outside normal storage sites when subcutaneous adipose tissue capacity is exceeded (for example to liver, heart, lungs, skeletal muscles), has been linked with worsening lung function loss in IPF [82] and may precede sarcopenia (diagnosed by low strength plus low muscle mass), constitute an exacerbated condition, or even be present independently of sarcopenia [83,84].It occurs through various mechanisms, including direct lipotoxicity [85], adipokine-mediated inflammation [86] and accumulation of immune cells in dysfunctional adipose tissue [87] along with insulin resistance that promotes pulmonary fibrosis through TGF-β signaling [88]. As a fact, greater fat infiltration correlates with worse outcomes; in fact, “for each doubling in pericardial adipose tissue volume, the odds of interstitial lung abnormalities increased by 20%” [89].

Likewise, pancreatic fat infiltration contributes to diabetes occurrence, a common comorbidity in IPF patients (30–35%) [90]. Although we did not find estadistical differences in our sample, in a cohort study, diabetes presence increased the risk of mortality in the IPF cohort by a factor of 2.5 [91]. This could be explained by diabetes itself, with consequent greater insulin resistance leading to catabolism-anabolism imbalance, resulting in greater metabolic waste that may accelerate the process toward cachexia.

To calculate myosteatosis in muscle mass at T12-CT, we applied the methodology described by Salhöfer et al., who demonstrated in a cohort of 79 IPF patients that the myosteatosis index constitutes an independent predictor of a 1.12-times greater risk of mortality, with significantly shorter median survival in patients with high myosteatosis compared to those with low myosteatosis (14 vs. 33 months). When applied to our cohort, the myosteatosis threshold of 36.46 HU at T12-CT showed excellent discriminative power to identify high-risk patients.

Moreover, our study identified clinically relevant diagnostic cut-off values for cachexia risk stratification, facilitating identification of patients requiring comprehensive assessment, among them, TUG cut-off > 8 s was significantly associated with a 7.85-fold increase in mortality risk, in contrast with the >20 s cut-off point established to determine severe dysfunctionality in EWGSOP2.

Finally, our findings support mitochondrial dysfunction in IPF cachexia pathogenesis. GDF-15 elevation reflects systemic stress and directly promotes cachexia through appetite suppression via the GFRAL receptor and muscle atrophy induction, as demonstrated by Verhamme et al. In our cohort, GDF-15 showed inverse correlations with cellular integrity markers while correlating positively with cachexia across all definitions. Conversely, reduced PGC-1α indicates mitochondrial dysfunction with impaired cellular energy production and was consistently lower in cachectic groups. Together, elevated GDF-15 and reduced PGC-1α create a metabolic phenotype characteristic of IPF cachexia, aligning with evidence from Amado et al. [92] demonstrating the diagnostic precision of mitochondrial peptides in chronic respiratory diseases.

This study has some limitations. First, the sample size was moderate, which may limit the strength of some findings, especially when comparing smaller subgroups. Second, most patients were male, which reflects the typical distribution of IPF, but reduces our ability to explore differences between sexes. Third, not all patients had a CT scan available at the T12 level, which limited the application of some cachexia definitions. In addition, while using T12 is a practical and validated alternative to L3, more studies are needed to confirm its accuracy for measuring muscle quality. Lastly, this was an observational and cross-sectional study, so we cannot establish causal relationships or track how cachexia progresses over time.

Future studies should include larger and more diverse patient populations, with a better balance of sexes. Long-term follow-up would help us understand how cachexia develops and whether it responds to treatment. External validation of the proposed cachexia criteria, especially those using T12-CT and mitochondrial biomarkers, is also needed. In addition, future work should explore targeted interventions—such as nutritional therapy, physical training, or anti-inflammatory and metabolic treatments—to see if early detection and management of cachexia can improve outcomes in IPF.

## 5. Conclusions

Cachexia is a common and clinically relevant condition in IPF, particularly when assessed using disease-specific criteria. In our cohort, the presence of the “IPF Cachexia Syndrome” criteria identified nearly half of the patients and was significantly associated with reduced survival, highlighting its potential value for early risk detection.

The integration of morphofunctional assessment, including body composition at the T12 level, functional performance tests, and mitochondrial biomarkers such as GDF-15 and PGC-1α, enabled a more accurate classification of cachexia phenotypes. Elevated GDF-15 levels, impaired TUG performance, and the presence of the IPF-specific criteria were all independently associated with increased mortality.

These results support the need for early detection and personalized management guided by nutritional phenotypes in IPF, which are consistent with current evidence, and may help improve long-term outcomes in this population.

## Figures and Tables

**Table 1:** Comparison of Morphofunctional and Biochemical Parameters According to Evans Criteria for Cachexia.

BC Technique	Variables	Cachexia Evans Criteria No (*n* = 64)	Cachexia Evans Criteria Yes (*n* = 21)	*p*-Value
	Weight	80.714 ± 12.992	74.029 ± 9.557	0.033
	Height	169.797 ± 7.927	166.381 ± 7.997	0.091
	BMI	27.900 ± 3.383	26.819 ± 3.683	0.217
	HGS	36.297 ± 8.544	22.552 ± 5.972	<0.001
	TUG	7.445 ± 2.141	8.491 ± 1.957	0.014
	GDF15	4156.029 ± 2800.271	3943.801 ± 2035.283	0.782
	PGC-1α	4.434 ± 2.592	3.788 ± 1.777	0.360
BIVA	Rz	515.358 ± 56.870	550.671 ± 72.981	0.024
	Xc	43.542 ± 6.849	45.776 ± 10.454	0.262
	FFM	56.295 ± 7.425	51.005 ± 6.429	0.004
	BCM	26.587 ± 5.026	23.700 ± 4.880	0.024
	TBW	42.041 ± 6.051	38.124 ± 4.985	0.009
	FM	24.419 ± 8.273	23.024 ± 7.561	0.496
	Pha	4.847 ± 0.713	4.762 ± 0.919	0.661
	NAK	1.166 ± 0.178	1.175 ± 0.217	0.847
	Hydration	74.598 ± 2.339	74.800 ± 2.617	0.745
	Nutrition	775.880 ± 166.136	715.381 ± 143.528	0.139
	Spha	−0.956 ± 0.859	−0.967 ± 1.211	0.964
NU	RF_CSA	3.453 ± 1.062	3.024 ± 0.686	0.155
	RF-Y-Axis	1.146 ± 0.289	1.022 ± 0.218	0.076
	L-SAT	0.789 ± 0.490	0.784 ± 0.635	0.973
	T-SAT	1.557 ± 0.682	2.049 ± 0.732	0.007
	S-SAT	0.689 ± 0.282	0.862 ± 0.312	0.014
	VAT	0.639 ± 0.312	0.691 ± 0.282	0.496
T12CT	SMI_T12CT	27.003 ± 6.903	24.093 ± 6.659	0.142
	Muscle_HU_T12CT	38.359 ± 7.606	40.750 ± 5.986	0.250
	VAT_area_T12CT	187.789 ± 87.540	149.270 ± 56.834	0.099

Mean ± SD are presented for each variable. *p*-values were obtained from independent samples *t*-test or Mann-Whitney U test for comparisons between groups with and without cachexia. Variables with a *p*-value < 0.05 indicate significant differences between groups. Abbreviations: BMI (Body Mass Index), Rz (Impedance Ratio), Xc (Reactance), FFM (Fat-Free Mass), BCM (Body Cell Mass), TBW (Total Body Water), FM (Fat Mass), Pha (Phase Angle), NAK (Normalized Amplitude of K), Spha (Standardized Phase Angle), RF_CSA (Cross-Sectional Area of the Rectus Femoris), RF-Y-Axis (Y-Axis of the Rectus Femoris), L-SAT (Lower-Limb Skeletal Area), T-SAT (Trunk Skeletal Area), S-SAT (Skeletal Area at Sacral Level), VAT (Visceral Adipose Tissue), HGS (Hand Grip Strength), TUG (Timed Up and Go Test), SMI_T12CT (Skeletal Muscle Index at T12), Muscle_HU_T12CT (Muscle Hounsfield Units at T12), VAT_area_T12CT (Visceral Fat Area at T12), GDF15 (Growth Differentiation Factor 15), PGC-1α (Peroxisome Proliferator-Activated Receptor Gamma Coactivator 1-Alpha), BC (body composition), BIVA (Bioelectrical Impedance Vector Analysis), NU (Nutritional Ultrasound), T12CT (computed tomography at T12 level).

**Table 2:** Comparison of Morphofunctional and Biochemical Parameters According to Martin Criteria for Cachexia.

BC Technique	Variables	Cachexia Martin Criteria No (*n* = 43)	Cachexia Martin Criteria Yes (*n* = 18)	*p*-Value
	Weight	80.43 ± 12.60	76.46 ± 15.42	0.298
	Height	169.37 ± 6.40	167.56 ± 10.49	0.410
	BMI	27.97 ± 3.64	27.09 ± 3.96	0.406
	HGS	34.77 ± 10.34	29.22 ± 9.13	0.053
	TUG	7.35 ± 1.81	7.63 ± 2.27	0.154
	GDF15	3552.46 ± 1380.68	5013.55 ± 3021.27	0.027
	PGC-1α	4.79 ± 2.50	3.35 ± 2.37	0.097
BIVA	Rz	502.86 ± 49.06	558.47 ± 52.81	0.001
	Xc	44.87 ± 7.07	42.39 ± 6.23	0.202
	FFM	57.12 ± 6.86	51.09 ± 6.72	0.003
	BCM	27.89 ± 4.89	22.38 ± 3.57	0.001
	TBW	42.45 ± 5.38	38.27 ± 5.21	0.007
	FM	23.30 ± 7.94	25.37 ± 10.65	0.408
	Pha	5.11 ± 0.72	4.34 ± 0.58	0.001
	NAK	1.14 ± 0.17	1.22 ± 0.17	0.085
	Hydration	74.28 ± 1.84	74.96 ± 2.41	0.143
	Nutrition	809.16 ± 180.84	673.82 ± 99.95	0.001
	Spha	−0.81 ± 0.96	−1.27 ± 0.85	0.059
NU	RF_CSA	3.57 ± 1.04	2.75 ± 0.63	0.003
	RF-Y-Axis	1.19 ± 0.32	1.01 ± 0.18	0.018
	L-SAT	0.74 ± 0.48	0.92 ± 0.68	0.186
	T-SAT	1.71 ± 0.76	1.80 ± 0.77	0.704
	S-SAT	0.72 ± 0.28	0.81 ± 0.35	0.429
	VAT	0.64 ± 0.28	0.64 ± 0.20	0.682
T12CT	SMI_T12CT	28.42 ± 6.59	20.86 ± 4.28	0.001
	Muscle_HU_T12CT	40.62 ± 7.06	35.22 ± 6.28	0.007
	VAT_area_T12CT	183.84 ± 92.24	160.85 ± 45.61	0.591

Abbreviations: BMI (Body Mass Index), Rz (Impedance Ratio), Xc (Reactance), FFM (Fat-Free Mass), BCM (Body Cell Mass), TBW (Total Body Water), FM (Fat Mass), Pha (Phase Angle), NAK (Normalized Amplitude of K), Spha (Standardized Phase Angle), RF_CSA (Cross-Sectional Area of the Rectus Femoris), RF-Y-Axis (Y-Axis of the Rectus Femoris), L-SAT (Lower-Limb Skeletal Area), T-SAT (Trunk Skeletal Area), S-SAT (Skeletal Area at Sacral Level), VAT (Visceral Adipose Tissue), HGS (Hand Grip Strength), TUG (Timed Up and Go Test), SMI_T12CT (Skeletal Muscle Index at T12), Muscle_HU_T12CT (Muscle Hounsfield Units at T12), VAT_area_T12CT (Visceral Fat Area at T12), GDF15 (Growth Differentiation Factor 15), PGC-1α (Peroxisome Proliferator-Activated Receptor Gamma Coactivator 1-Alpha), BC (body composition), BIVA (Bioelectrical Impedance Vector Analysis), NU (Nutritional Ultrasound), T12CT (computed tomography at T12 level).

**Table 3:** Comparison of Morphofunctional and Biochemical Parameters According to Idiopathic Pulmonary Fibrosis Cachexia Syndrome (Proposed Criteria).

BC Technique	Variables	IPF Cachexia Syndrome No (*n* = 49)	IPF Cachexia Syndrome Yes (*n* = 36)	*p*-Value
	Weight	79.563 ± 11.715	78.381 ± 13.680	0.670
	Height	169.959 ± 7.121	167.583 ± 9.057	0.179
	BMI	27.496 ± 3.284	27.819 ± 3.745	0.674
	HGS	33.645 ± 10.324	31.889 ± 9.441	0.424
	TUG	7.663 ± 2.095	7.734 ± 2.216	0.390
	GDF15	4046.193 ± 2805.247	4187.154 ± 2344.455	0.472
	PGC-1α	4.585 ± 2.568	3.790 ± 2.121	0.163
BIVA	Rz	514.933 ± 64.246	536.536 ± 59.085	0.117
	Xc	44.363 ± 8.113	43.728 ± 7.657	0.716
	FFM	56.233 ± 7.477	53.294 ± 7.329	0.075
	BCM	26.869 ± 5.407	24.519 ± 4.413	0.036
	TBW	42.080 ± 6.061	39.703 ± 5.771	0.072
	FM	23.331 ± 7.062	25.086 ± 9.301	0.325
	Pha	4.945 ± 0.823	4.664 ± 0.653	0.094
	NAK	1.168 ± 0.213	1.167 ± 0.146	0.081
	Hydration	74.771 ± 2.638	74.471 ± 2.026	0.656
	Nutrition	799.884 ± 150.073	707.917 ± 164.940	0.009
	Spha	−0.861 ± 1.002	−1.092 ± 0.870	0.390
NU	RF_CSA	3.580 ± 1.023	3.030 ± 0.875	0.013
	RF-Y-Axis	1.165 ± 0.300	1.049 ± 0.230	0.056
	L-SAT	0.701 ± 0.352	0.903 ± 0.683	0.495
	T-SAT	1.696 ± 0.690	1.655 ± 0.773	0.676
	S-SAT	0.730 ± 0.284	0.737 ± 0.321	0.946
	VAT	0.660 ± 0.344	0.640 ± 0.240	0.875
T12CT	SMI_T12CT	28.774 ± 7.689	23.852 ± 5.200	0.005
	Muscle_HU_T12CT	44.252 ± 5.624	34.288 ± 4.870	<0.001
	VAT_area_T12CT	176.204 ± 78.810	177.825 ± 85.279	0.891

Abbreviations: BMI (Body Mass Index), Rz (Impedance Ratio), Xc (Reactance), FFM (Fat-Free Mass), BCM (Body Cell Mass), TBW (Total Body Water), FM (Fat Mass), Pha (Phase Angle), NAK (Normalized Amplitude of K), Spha (Standardized Phase Angle), RF_CSA (Cross-Sectional Area of the Rectus Femoris), RF-Y-Axis (Y-Axis of the Rectus Femoris), L-SAT (Lower-Limb Skeletal Area), T-SAT (Trunk Skeletal Area), S-SAT (Skeletal Area at Sacral Level), VAT (Visceral Adipose Tissue), HGS (Hand Grip Strength), TUG (Timed Up and Go Test), SMI_T12CT (Skeletal Muscle Index at T12), Muscle_HU_T12CT (Muscle Hounsfield Units at T12), VAT_area_T12CT (Visceral Fat Area at T12), GDF15 (Growth Differentiation Factor 15), PGC-1α (Peroxisome Proliferator-Activated Receptor Gamma Coactivator 1-Alpha), BC (body composition), BIVA (Bioelectrical Impedance Vector Analysis), NU (Nutritional Ultrasound), T12CT (computed tomography at T12 level).

**Table 4:** Comprehensive table of nutritional phenotypes according to different criteria.

Nutritional Phenotype	Criteria	Counts (*n*)	% of Total
Cachexia, Evans’ criteria		21	24.7%
	Lost weight > 5% or BMI < 20 kg/m^2^	47	55.3%
	Low-Intake	47	55.3%
	Inflammation (CRP > 5 mg/dL)	35	41.2%
	Low FFMI (<17 kg/m^2^ for men or <15 kg/m^2^ for women)	8	9.4
	Low muscle strength (<27 kg for men or <16 kg for women)	19	22.4
Cachexia, Martin’s criteria		18	29.5%
	Lost weight > 5%	47	55.3%
	Myosteatosis (IMAT > 15.25%)	34	55.7%
	Low muscle mass by T12CT (SMI ≤ 28.8 cm^2^/m^2^)	44	72.1%
IPF Cachexia Syndrome criteria		36	42.4%
	Lost weight > 5%	47	55.3%
	Inflammation (CRP > 5 mg/dL)	35	41.2%
	Low muscle mass by T12TC (SMI ≤ 24.5 cm^2^/m^2^) Low muscle mass by ASMI from BIVA (<7 kg/m^2^ for men or <5.5 kg/m^2^ for women)	33 55	54.1% 64.7%
	Myoesteatosis HU muscle (<36.46 UH)	25	41.0%

Abbreviations: CRP, C-reactive protein; ASMI, Appendicular Skeletal Muscle Index; BMI, body mass index; FFMI, fat-free mass index, IMAT, intramuscular adipose tissue SMI, Skeletal Muscle Index; HU, Hounsfield Units; T12TC, Computed Tomography at T12 vertebral level.

**Table 5:** Diagnostic performance of individual morphofunctional and biochemical predictors for the identification of IPF cachexia syndrome.

Variable	Cut-Off	Sensibility	Especificity	PPV	NPV	AUC (IC 95%)	*p*-Value
Pha	4.8	75.0%	64.3%	60.0%	78.3%	0.69 (0.538–0.843)	<0.001
SPhA	−1.56	37.9%	89.5%	75.0%	48.6%	0.587 (0.424–0.75)	<0.001
RF_CSA	3.0	55.2%	78.9%	80.0%	53.6%	0.652 (0.481–0.822)	<0.001
TUG	8.0	50.0%	78.6%	62.5%	62.5%	0.646 (0.481–0.812)	<0.001
GDF15	4412.0	44.8%	94.7%	92.9%	52.9%	0.632 (0.473–0.79)	<0.001

Abbreviations: Pha, phase angle; SPhA, standardized phase angle; RF_CSA, rectus femoris cross-sectional area; TUG, Timed Up and Go test; GDF15, growth differentiation factor 15; AUC, area under the curve; PPV, positive predictive value; NPV, negative predictive value.

**Table 6:** Univariate and Multivariate Analysis of Mortality Predictors.

Dependent: Mortality		0	1	OR (Univariable)	OR (Multivariable)
GDF15_CachexiaSyndrome	0	37 (88.1)	5 (11.9)	-	-
	1	12 (60.0)	8 (40.0)	4.93 (1.39–19.22, *p* = 0.016)	4.62 (1.10–22.72, *p* = 0.043)
TUG_CachexiaSyndrome	0	37 (88.1)	5 (11.9)	-	-
	1	12 (60.0)	8 (40.0)	4.93 (1.39–19.22, *p* = 0.016)	7.85 (1.53–50.79, *p* = 0.019)
Sexo	0	9 (90.0)	1 (10.0)	-	-
	1	40 (76.9)	12 (23.1)	2.70 (0.44–52.35, *p* = 0.368)	4.13 (0.45–100.45, *p* = 0.269)
Age	Mean (SD)	70.9 (7.5)	74.5 (4.2)	1.09 (0.99–1.22, *p* = 0.103)	1.03 (0.91–1.20, *p* = 0.624)
BMI	Mean (SD)	27.9 (3.7)	27.2 (3.2)	0.95 (0.79–1.13, *p* = 0.563)	0.90 (0.70–1.12, *p* = 0.372)

Abbreviatures: GDF15: Growth Differentiation Factor 15; TUG: Timed Up and Go; BMI: Body Mass Index; OR: Odds Ratios; SD: Standard Deviation.

## Data Availability

The original contributions presented in the study are included in the article, further inquiries can be directed to the corresponding authors.

## Supplementary Materials

The following supporting information can be downloaded at: https://www.mdpi.com/article/10.3390/nu17172739/s1, Figure S1. Flow chart diagram of patient’s selection in our study; Figure S2. ROC-curve for muscle attenuation (Hounsfield Units) at T12 to detect myosteatosis (defined as >15% IMAT); Figure S3: individual ROC curve.

## Abbreviations

The following abbreviations are used in this manuscript:

ASMI	Appendicular Skeletal Muscle Index
ATS/ERS/JRS/ALAT	American Thoracic Society/European Respiratory Society/Japanese Respiratory Society/Latin American Thoracic Association
AUC	Area Under the Curve
BC	Body Composition
BCM	Body Cell Mass
BIVA	Bioelectrical Impedance Vector Analysis
BMI	Body Mass Index
CI	Confidence Interval
COPD	Chronic Obstructive Pulmonary Disease
CRP	C-Reactive Protein
CT	Computed Tomography
DLCO	Diffusion Capacity of the lung for Carbon Monoxide
EWGSOP2	European Working Group on Sarcopenia in Older People 2
FFM	Fat-Free Mass
FM	Fat Mass
FVC	Forced Vital Capacity
GDF-15	Growth Differentiation Factor 15
HGS	Hand Grip Strength
HR	Hazard Ratio
HU	Hounsfield Units
IMAT	Intramuscular Adipose Tissue
IPF	Idiopathic Pulmonary Fibrosis
MFA	Morphofunctional Assessment
NU	Nutritional Ultrasound
NAK	Normalized Amplitude of K
OR	Odds Ratio
Pha	Phase Angle
PGC-1α	Peroxisome Proliferator-Activated Receptor Gamma Coactivator 1-Alpha
RF_CSA	Rectus Femoris Cross-Sectional Area
RF-Y-Axis	Y-Axis of the Rectus Femoris
ROC	Receiver Operating Characteristic
SAT	Subcutaneous Adipose Tissue
SD	Standard Deviation
SMI	Skeletal Muscle Index
SMI_T12CT	Skeletal Muscle Index at T12
SPha	Standardized Phase Angle
T12CT	Computed Tomography at T12 vertebral level
TBW	Total Body Water
TUG	Timed Up and Go test
VAT	Visceral Adipose Tissue
VPN	Negative Predictive Value
