# Supplementary material for: Cachexia Phenotyping Through Morphofunctional Assessment and Mitocondrial Biomarkers (GDF-15 and PGC-1α) in Idiopathic Pulmonary Fibrosis
Source: Nutrients. 2025 Aug 24;17(17):2739. doi: 10.3390/nu17172739 (PMC12430740; doi:10.3390/nu17172739)
Supplement: Supplementary file 1 [file nutrients-17-02739-s001.zip › nutrients-3791957-supplementary.pdf]

SUPPLEMENTARY

Figure S1. Flow chart diagram of patient's selection in our study.

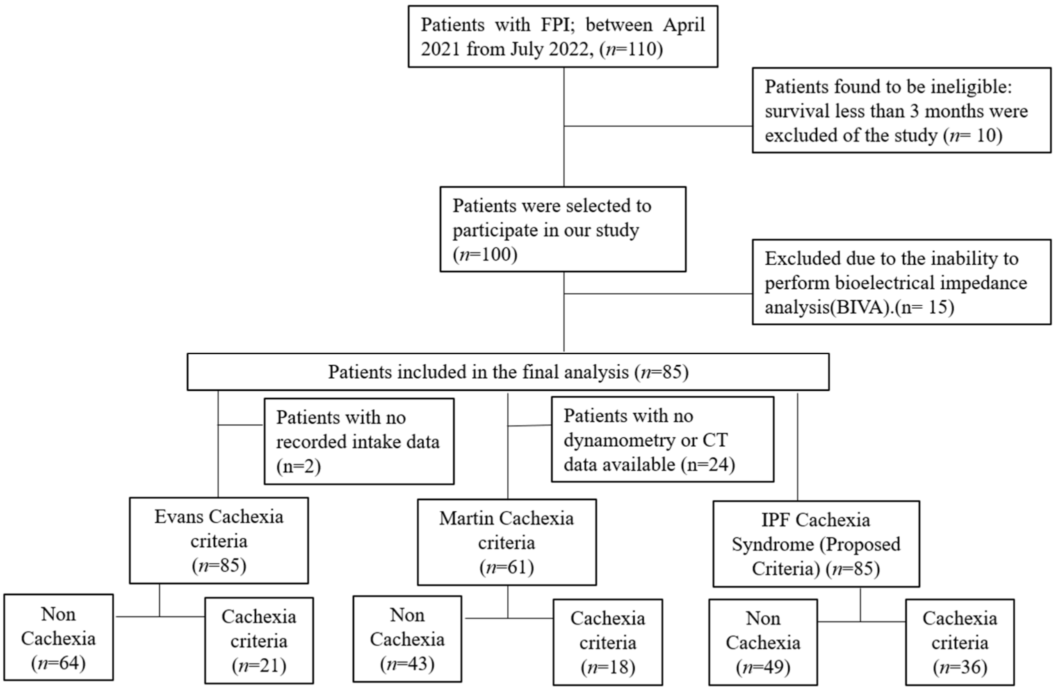

Abbreviations: IPF idiopathic pulmonary fibrosis; CT computed tomography.

Figure S2. ROC-curve for muscle attenuation (Hounsfield Units) at T12 to detect myosteatosis (defined as >15% IMAT).

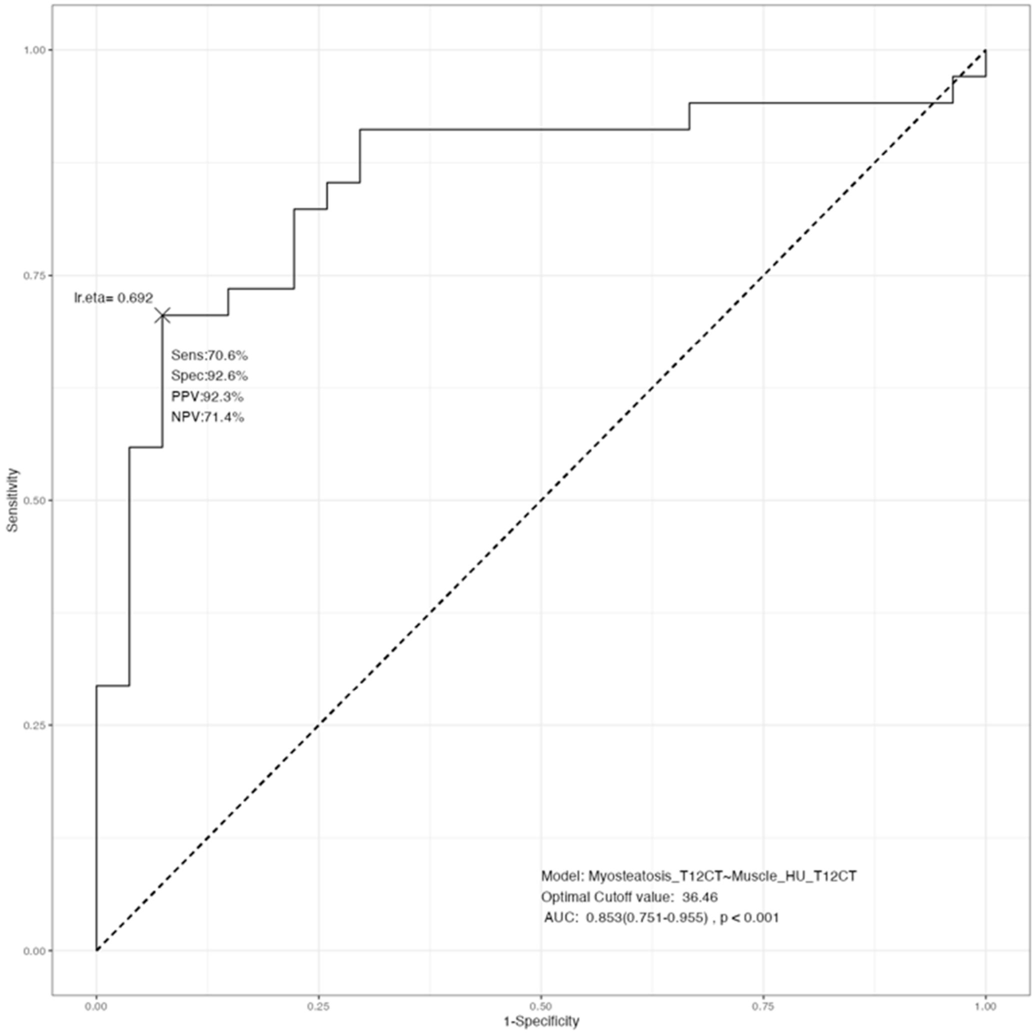

Abbreviations: Sens (sensibility), spec (specificity), PPV (positive predictive value), NPV (negative predictive value), AUC (area under the curve), ROC (receiver operating characteristic), CT (computed tomography), HU (hounsfield units).

Figure S3: individual ROC curve.

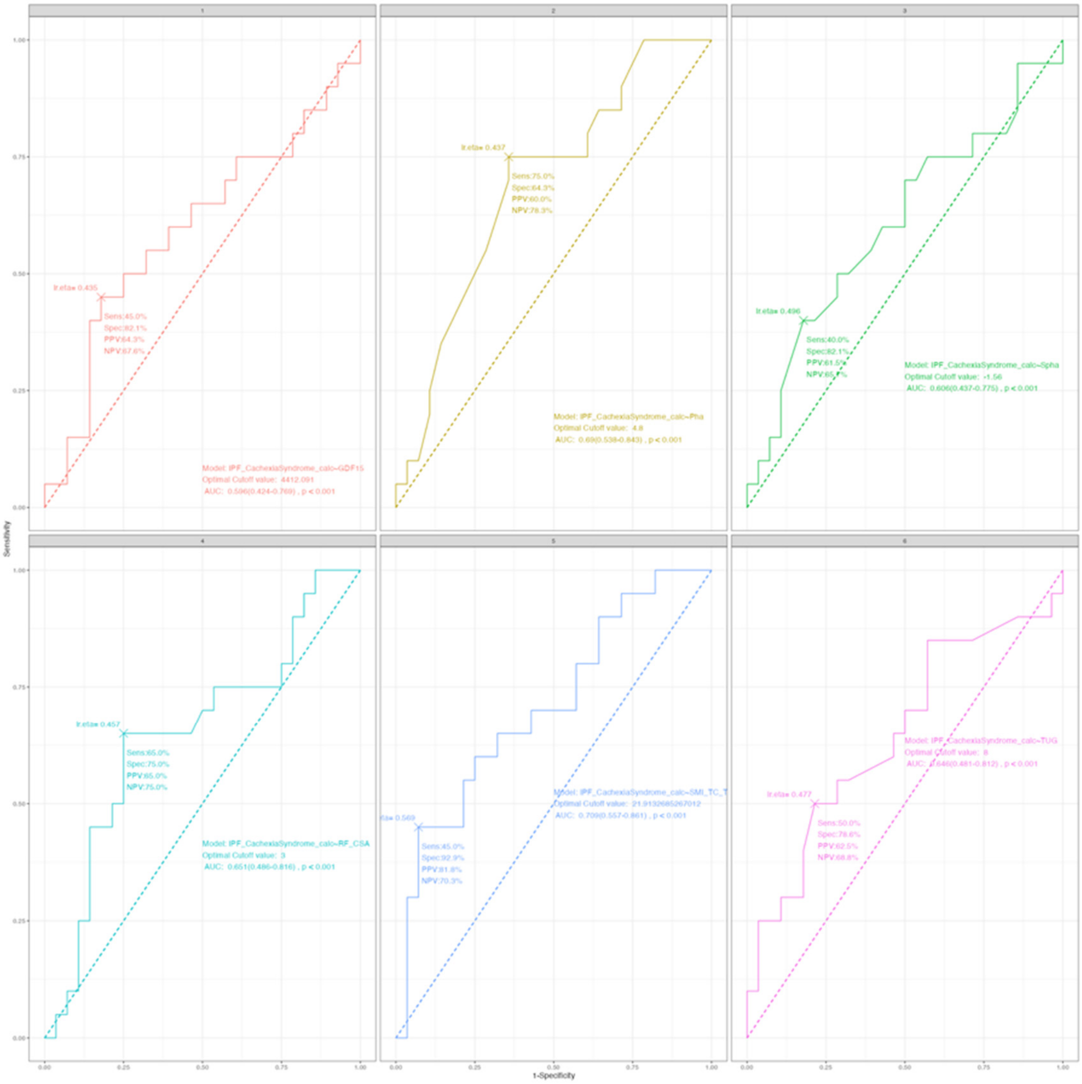

Abbreviations: Sens (sensibility), spec (specificity), PPV (positive predictive value), NPV (negative predictive value), AUC (area under the curve), ROC (receiver operating characteristic).
